# Accuracy of Voluntary Force Modulation During the Isometric Mid-Thigh Pull

**DOI:** 10.3390/sports14020083

**Published:** 2026-02-14

**Authors:** S. Alexander Long, Olivia Vadas, Stephanie Balint, Michael H. Stone, Christopher B. Taber

**Affiliations:** 1 Department of Physical Therapy and Human Movement Science, Sacred Heart University, Fairfield, CT 06825, USA; 2 Frank H. Netter School of Medicine, Quinnipiac University, North Haven, CT 06473, USA; 3 Center of Excellence for Sport Science and Coach Education, Department of Sport, Exercise, Recreation, and Kinesiology, East Tennessee State University, Johnson City, TN 37614, USA

**Keywords:** muscle, strength, technical, precision, control, anchor

## Abstract

The purpose of this study was to investigate voluntary force modulation accuracy during the isometric mid-thigh pull (IMTP) and to investigate biological sex and relative strength as factors relating to error. Strength-trained males (n = 18) and females (n = 18) completed ascending (ASC) (25%, 50%, 75%) or descending (DESC) (75%, 50%, 25%) submaximal testing followed by maximal testing. Subjects rested before completing the opposite submaximal testing sequence. External feedback was not provided during testing. Measured and intended (INT) forces were analyzed with two-way repeated-measures ANOVAs with within- (ASC, DESC, and INT) and between-subject factors (male or female). Independent-samples *t*-tests analyzed differences in error between males and females. Pearson correlations were calculated to investigate associations between relative strength and error. Statistically significant differences were observed between INT and measured force at every intensity (*p* < 0.05); however, differences in error were not significant between males and females (*p* > 0.05). Statistically non-significant small relationships were observed between relative strength and error (*p* > 0.05). Subjects demonstrate error in force modulation during the IMTP, with the greatest error occurring at lower relative intensity. However, these results indicate that biological sex and relative strength may not influence force modulation accuracy.

## 1. Introduction

Maximal muscular force is strongly related to performance in a wide range of general sporting movements [1,2,3,4,5,6,7,8], indicating that muscular strength is likely an important factor in achieving success in athletic competition. These associations have contributed to the widespread inclusion of strength training and testing among athletic populations. In the assessment of muscular strength, isometric testing has yielded excellent measurement reliability [9]. Specifically, the isometric mid-thigh pull (IMTP), a bilateral, multi-joint test that emulates body positioning at the beginning of the second pull of the snatch and clean, is often used to assess isometric peak force (IPF) during maximal-effort testing [2,4,7,8,10,11]. The IMTP test has been recommended for assessing muscular strength, as it may be more informative, less fatiguing, and pose a lower injury risk than traditional repetition-maximum testing [2].

Despite observed associations between muscular strength and general sport skill performance, simple observation and empirical evidence indicate that the execution of sport-specific technical skills is not explained solely by force capacity. Weak-to-moderate and equivocal associations have been reported between maximal muscular strength and technical sport-specific skill performance, including, but not limited to, golf handicap [12] and putting accuracy [13] and basketball shooting performance [14,15]. Precise expression of submaximal muscular force is required in the execution of sport-specific technical movements, suggesting that the ability to modulate muscular force may be more critical than force capacity. Unlike maximal muscular strength, which has been extensively investigated in athletic populations, the ability to modulate voluntary muscular force has been less studied [16,17,18], leading to a poor understanding of potential associations with force capacity and precluding the determination of optimal methods for testing and monitoring this ability in athletic populations.

Muscular force generation is primarily dependent upon motor unit recruitment and modulation of motor unit firing rates (rate coding) [19,20,21], which initiate and modulate actomyosin interaction through the excitation–contraction coupling mechanism [22,23]. During a maximal voluntary contraction (MVC), it is expected that the maximum number of motor units is recruited [19] and motor unit firing rates are greatest [24]. In contrast, modulation of submaximal force is achieved by recruiting only those motor units necessary to generate and maintain a magnitude of intended force, while rate coding generally occurs at lower frequencies, improving force steadiness [20]. Compared to performing an MVC, modulation of submaximal force may be relatively more complex, as accuracy in intended force is also dependent upon individual perception [18,25]. It has been suggested that those who practice strength training may be able to modulate voluntary muscular force with greater accuracy as a consequence of neurological and morphological adaptations, which may enhance force production and perception mechanisms [18,21,26].

Indeed, the results of recent investigations indicate that trained athletes demonstrate greater accuracy in voluntary force modulation compared to untrained controls [18,26]. However, investigations of force modulation accuracy focusing on strength-trained subjects and athletes have yielded inconsistent results. Keller et al. [16] reported that subjects overestimated force at very low relative intensity (i.e., 10% MVC) and underestimated force at very high relative intensity (i.e., 90% MVC), with the greatest accuracy observed at 50% MVC. In contrast, Miyamoto et al. [17] and Rizzato et al. [18] reported that trained athletes were most accurate at low relative intensities (i.e., 20% and 25% MVC), with error increasing with effort intensity. These incongruent results are not easily explained and confound an understanding of the force modulation capabilities expected of strength-trained populations.

Disparities in force modulation ability have also been reported when subject biological sex and chronological age were considered. When investigating force modulation accuracy relative to subject MVC, Keller et al. [16] reported sex-based disparities between strength-trained males and females, where females demonstrated greater accuracy at low (e.g., 30% MVC) and very high (e.g., 90% MVC) relative intensities [16]. Recent evidence indicates that individual differences in rapid force development are likely attributed to disparities in relative strength, rather than biological sex [27]. However, it does not appear that subject relative strength has been considered as a factor influencing voluntary force modulation. Recently, voluntary force modulation ability was investigated in younger and older adults, with researchers reporting greater variability and poorer accuracy in the latter [28]. The ability to voluntarily modulate muscular force is critical to the performance of activities of daily living, maintaining independence, and mitigating injury risk associated with falls as adults age [29]. Therefore, the assessment of voluntary force modulation may be an indicator of health as well as athletic ability, with relevance for younger and older adults and untrained and athletic populations.

The methods of testing used in previous research may be an important consideration, as prior investigators assessed force modulation capabilities using unilateral testing, isolating musculature acting at a single joint [16,17,18,26]. As these actions do not emulate sporting movements, which are largely compound and bilateral, the force modulation capabilities reported in prior research may not reflect athlete capabilities during competition. It is possible that the assessment of force modulation ability during the IMTP may improve the ecological validity of test results, providing greater insight into this crucial aspect of athletic performance.

The purpose of this study was to investigate force modulation accuracy during the IMTP test when performed by subjects with prior strength training experience. Comparisons were made between male and female subjects to examine potential disparities in error. Associations between subject relative strength and force modulation error were examined to investigate the relevance of muscular strength to force modulation ability. It was hypothesized that subjects would overestimate force at 25%, would be accurate at 50%, and would underestimate force at 75% relative intensity of effort; that female subjects would be more accurate in modulating force at low (i.e., 25%) and high (i.e., 75%) relative intensities; and that subject relative strength would be strongly associated with force modulation accuracy at every relative intensity investigated.

## 2. Methods

### 2.1. Experimental Approach to the Problem

**Figure 1:**
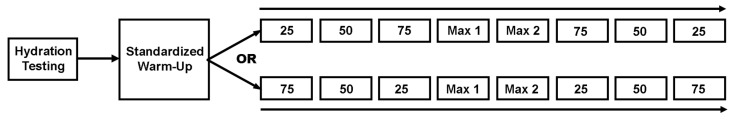
Overview of study procedures. Subjects were instructed to express 25 = 25%, 50 = 50%, or 75 = 75% of maximum effort during ascending (ASC) and descending (DESC) trials. ASC and DESC trials were completed in a randomized and counterbalanced manner. The arrows indicate the direction of progression during testing. Max 1 = first maximal-effort trial; Max 2 = second maximal-effort trial.

A randomized, repeated-measures, and counterbalanced design was employed to investigate the accuracy of voluntary modulation of muscular force during the IMTP test. Subjects reported for a single testing session in which multiple submaximal- and maximal-effort IMTP trials were performed. Between-subject comparisons were conducted to assess the force modulation accuracy of males and females. Subjects were first randomly assigned to complete submaximal testing in ascending (ASC) (25%, 50%, 75%) or descending order (DESC) (75%, 50%, 25%), after which subjects completed maximal-effort testing. Following maximal testing, subjects completed the opposite submaximal testing sequence to which they were initially assigned. Subjects were not provided external feedback during testing sessions. Net IPF, determined through maximal-effort testing, was used to calculate the intended force (INT) at each relative intensity investigated (INT25, INT50, and INT75). An overview of study procedures is presented in Figure 1.

### 2.2. Subjects

A priori power analysis was conducted to identify an appropriate sample size with the intention of detecting at least a moderate effect, as moderate-to-large effects have been reported previously when similar interaction effects were investigated [16]. User input variables were as follows: effect size f: 0.25; α error probability: 0.05; power (1-β error probability): 0.80; number of groups: 2; number of measurements: 3; correlation among repeated measures: 0.5; nonsphericity correction ε: 1 (G*Power, version 3.1.9.7, Heinrich-Heine-Universität Düsseldorf, Düsseldorf, Germany). The analysis yielded a recommended sample size of 28.

Males (n = 18) and females (n = 18) with a minimum of one year of strength training experience including weightlifting derivatives (average training frequency = 4.1 ± 1.2 days/week) volunteered to participate in this study. Subject descriptive statistics are presented in Table 1. Subjects were invited to participate through word of mouth. Inclusion criteria included a minimum of one year of resistance training experience including weightlifting derivatives. Subjects were excluded from participating in this study if they reported any musculoskeletal injury in the past six months which would affect their ability to perform the dynamic mid-thigh pull exercise. When asked about prior experience performing the IMTP test, 24 of the 36 participants (10 males and 14 females) reported no prior experience. Prior to data collection, participants were informed of the study’s purpose, procedures, and risks of participation. Subjects indicated their willingness to participate by reading and signing a written informed consent document. This study received approval from the university’s institutional review board (protocol #: IRB-FY2025-99) and was conducted in accordance with the Declaration of Helsinki.

### 2.3. Procedures

#### 2.3.1. Hydration Testing and Anthropometrics

Prior to completing their scheduled testing session, subjects were instructed to refrain from physical exercise for a minimum of 24 h. Upon arrival at the laboratory, subjects completed a hydration assessment via refractometry (Atago, PAL-40S, Tokyo, Japan) to determine urine specific gravity (USG), which was used to assess hydration status and to mitigate dehydration effects on maximal physical performance during subsequent IMTP testing. Subjects provided one urine sample, which was assessed twice by a researcher. Subject hydration status was deemed acceptable when the two measured USG values from the same sample were within 0.002 and when both measured values were <1.020 [30]. If measured USG values were ≥1.020, the subject was instructed to drink water and wait at least 20 min before providing a second urine sample for testing. Following successful completion of hydration testing, the subjects’ heights were measured using a stadiometer (Seca 213, Hamburg, Germany), and subject body mass was determined using a dual force plate system (VALD, ForceDecks, Newstead, Australia).

#### 2.3.2. Standardized Warm-Up

Subjects completed a standardized dynamic warm-up consisting of 30 jumping jacks, followed by one set of five repetitions of the dynamic mid-thigh pull (MTP) exercise from the power position with an empty barbell (20 kg) (Pendlay, Nexgen, Fort Mill, SC, USA), followed by three sets of five repetitions of the dynamic MTP performed with a barbell loaded to either 40 kg (females) or 60 kg (males). All subjects indicated prior experience performing weightlifting derivatives, and they were coached during the dynamic MTP by a strength and conditioning professional certified by the National Strength and Conditioning Association. Immediately after completing the warm-up, while subjects assumed the power position, the distance from the floor to the bottom of the empty barbell was recorded with a meter stick to assist in establishing the appropriate height of an immovable bar used during subsequent IMTP testing.

#### 2.3.3. Procedures for Submaximal and Maximal IMTP Testing

**Figure 2:**
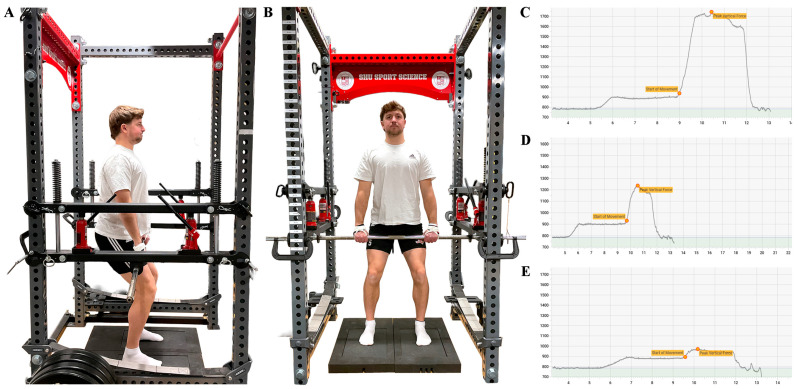
Isometric mid-thigh pull (IMTP) testing setup and example force–time curves. The IMTP testing setup is depicted from the lateral (**A**) and anterior view (**B**). Force–time curves were collected to identify gross isometric peak force during testing at 75% (**C**), 50% (**D**), and 25% (**E**) relative intensity of effort, with force data (N) presented on the vertical axis and time (seconds) presented on the horizontal axis. Net isometric peak force was calculated from force–time data and used in subsequent analysis. The initial rise in the force–time curve depicts the application of minimal force (i.e., 100 ± 100 N) to remove the slack from the system. Note that the individual pictured was not participating in this study, and the force–time curves depicted do not reflect the efforts of the individual. Data depicted were collected during pilot testing and were not included in the analysis.

A customized modified power rack (Sorinex, Lexington, SC, USA) positioned over dual force plates sampling at 1000 Hz (VALD, ForceDecks, Newstead, Australia) was adjusted for each subject so that an immovable bar was positioned approximately at the subject’s mid-thigh using the barbell height measured previously during the standardized warm-up. Subjects were instructed to hold onto the immovable bar and assume the power position, characterized by an upright torso, a knee angle of approximately 125° to 135°, and a hip angle of 140° to 150°, both verified via goniometry, and with the thighs in contact with the bar [2] (Figure 2A,B). Subjects were asked to confirm that they “felt strong in this position”. The subject’s hands were then secured to the immovable bar using weightlifting straps and athletic tape to eliminate grip strength as a limiting factor during testing. Immediately prior to the onset of each trial, subjects were instructed to gently pull against the immovable bar, expressing minimal force while producing steady tension against the bar to remove the slack from the system [2]. It has been suggested that ≤50 N of force should be applied to remove the slack from the system [2]. However, the application of such a trivial force was not sufficient to lift the 20 kg bar to make contact with the modified power rack used during testing. Pilot testing was conducted in which it was determined that 100 ± 100 N of force was required to remove the slack from the system prior to each trial. Subjects performed the IMTP test by pulling against the immovable bar and driving their feet into dual force plates positioned on the floor beneath them. The onset of each trial was detected using proprietary software (VALD, ForceDecks, Newstead, Australia) to identify the moment yank deviated from zero (first derivation method) [31] in unfiltered raw data [32]. To mitigate fatigue accumulation within and across trials, each trial was terminated when gross isometric peak force (absolute force expressed into the force plates including the force of body mass) plateaued (±100 N) or began to decrease (Figure 2C–E), resulting in trials typically lasting one to three seconds in duration.

Subjects completed two familiarization trials of the IMTP test at 50% perceived effort with 60 s of rest between trials. Following the familiarization trials, subjects rested for 60 s before completing six submaximal trials of the IMTP. Two submaximal-effort trials were each performed at 25%, 50%, and 75% perceived effort. During each respective trial, subjects were instructed to “pull with exactly 25% of your maximal effort”, “pull with exactly 50% of your maximal effort”, or “pull with exactly 75% of your maximal effort”. Subjects were given a countdown of “3, 2, 1” immediately before the start of each trial. Verbal encouragement was provided during each attempt of the IMTP test; however, subjects were not provided external feedback during the testing session. Submaximal attempts were completed in ASC (25%, 50%, 75%) and DESC (75%, 50%, 25%) order, with 60 s of rest between trials. The initial sequence of submaximal testing was randomly assigned to the first subject, after which the sequence for each subject alternated between ASC and DESC. After completion of the initial submaximal testing sequence, subjects rested passively for two minutes before completing maximal-effort testing. Following the completion of maximal testing, subjects rested passively for two minutes before completing submaximal testing in the opposite sequence to that initially assigned.

Identical procedures were used during each submaximal and maximal IMTP test, except for the intended level of effort and the duration of passive rest provided. Prior to each maximal-effort attempt, subjects were instructed to “pull as fast and as hard as possible during testing”. To mitigate fatigue accumulation, each maximal-effort trial was terminated when gross IPF plateaued (±100 N) or when gross IPF began to decrease, resulting in trials typically lasting one to three seconds in duration. Maximal-effort testing concluded when the gross IPF measured during two attempts was within ± 100 N, or when the subject performed a total of three attempts. Maximal-effort trials were separated by two minutes of passive rest.

### 2.4. Calculation of Intended Net IPF and Relative Error

Net IPF was calculated using proprietary software (VALD, ForceDecks, Newstead, Australia) in which the force of subject body mass, determined using the dual force plates system (VALD, ForceDecks, Newstead, Australia), was subtracted from gross IPF values recorded during submaximal and maximal-effort IMTP testing. Net IPF was considered to ensure that only the force perceived by each subject was included in the analysis of force modulation accuracy. The mean Net IPF achieved during the two closest attempts at maximal-effort testing was used to calculate the intended (INT) force values for each relative intensity by multiplying the mean Net IPF by 0.25 (INT25), 0.50 (INT50), and 0.75 (INT75). Error in force modulation was quantified as the difference between the Net IPF measured during each trial and the calculated INT force. Error was expressed as a percentage of the Net IPF achieved during maximal testing [relative error = ((measured Net IPF − INT Net IPF)/measured maximal Net IPF) × 100]. The relative error was calculated during ASC and DESC trials at each relative intensity investigated. The relative error was then averaged across ASC and DESC trials (average relative error). Subject relative strength was calculated as gross IPF allometrically scaled to body mass (IPFa) (N/kg^0.67^). Allometric scaling of force characteristics to body mass taken to the power of 0.67 has been recommended when normalizing force measurements for lean athletic populations [33], as such methods may account for the curvilinear relationship that has been observed between maximal strength and body mass [4,34].

### 2.5. Statistical Analysis

Data normality and sphericity were assessed using Shapiro–Wilk and Mauchly’s tests, respectively. Shapiro–Wilk testing indicated that the assumption of normality was met for all data (*p* > 0.05). Mauchly’s test showed that force values recorded and calculated at 75% relative intensity violated the assumption of sphericity (*p* < 0.01). Therefore, Greenhouse–Geisser correction was applied at the omnibus level during the analysis. Reliability of isometric force data was assessed using two-way mixed-model intraclass correlation coefficients (ICC_3,1_) [35] and the percentage coefficient of variation (CV) with 95% confidence intervals. Bland–Altman plots were created to examine agreement between measurements recorded during ASC and DESC trials. The ICC_3,1_ values were interpreted based on the lower bound 95% CI as <0.5—poor; 0.5 to 0.75—moderate; 0.75 to 0.90—good; and >0.90—excellent [35]; and CV values < 5% were interpreted as good, 5% to 10% as moderate, and >10% as poor [36].

To investigate the effects of trial and subject biological sex on force modulation accuracy, two-way repeated-measures ANOVAs were conducted with within- [trial (ASC, DESC, and INT)] and between-subject factors [sex (male or female)] for each relative intensity investigated (25%, 50%, and 75%). When the main effect of trial or trial × sex interaction effects were statistically significant, post hoc testing was conducted with Bonferroni adjustment. To identify potential effects of testing order, follow-up two-way ANOVAs with within- [trial (ASC and DESC)] and between-subject factors [testing order (ASC or DESC performed after maximal testing)] were conducted for the relative error observed at each relative intensity of effort. When trial × testing order interaction effects were statistically significant, post hoc testing was conducted with Bonferroni adjustment. Independent-samples *t*-tests were used to analyze differences in average relative error between males and females at each intensity. Effect sizes (Hedges’ *g*) were calculated for within-subject comparisons between ASC and DESC trials and the calculated INT force at each relative intensity. Effect sizes were interpreted as: <0.2—trivial; 0.2–0.59—small; 0.60–1.19—moderate; and >2.00—large [37]. To investigate associations between subject relative strength (IPFa) and average relative error in force modulation, Pearson’s product-moment correlation coefficients (*r*), 95% confidence intervals with Fisher z transformation [38], and coefficient of determination (*R*^2^) were calculated. Correlation coefficients were interpreted as: <0.1—trivial; 0.1 to 0.29—small; 0.3 to 0.49—moderate; 0.5 to 0.69—large; 0.7 to 0.89—very large; and ≥0.9—extremely large [37]. Standard calculations and figures were conducted and created using Microsoft Excel (Microsoft Corporation, Redmond, WA, USA, 2024). All statistical analyses were conducted using JASP (version 0.19.3; JASP team, 2025). The critical alpha level for all analyses was set at 0.05. Effect sizes were calculated, and correlation plots were created using R (version 4.5.1, R Core Team, Vienna, Austria, 2025).

## 3. Results

### 3.1. Reliability of Net Isometric Peak Force Measurements

**Figure 3:**
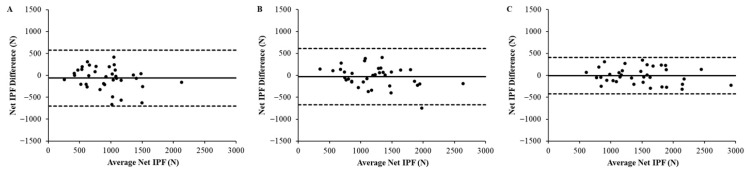
Bland–Altman plots depicting net isometric peak force measurement agreement between ascending and descending trials. Dashed lines indicate the upper and lower 95% limits of agreement. Bland–Altman plots indicated that measurement bias was 63 N, 31 N, and 6 N for trials performed at 25% (**A**), 50% (**B**), and 75% (**C**), respectively.

The ICC_3,1_ and CV values and 95% confidence intervals for isometric force data are presented in Table 2. The measurement reliability of isometric force data for the total sample, for male subjects, and for female subjects was excellent (ICC_3,1_: 0.951 to 0.989) and good (CV: 1.5% to 1.9%) during maximal testing, poor to moderate (ICC_3,1_: 0.328 to 0.726) and moderate (CV: 7.1% to 9.7%) when testing at 25% relative intensity, moderate to good (ICC_3,1_: 0.545 to 0.828) and moderate (CV: 6.4% to 6.9%) at 50% relative intensity, and moderate to excellent (ICC_3,1_: 0.668 to 0.909) and good to moderate (CV: 4.7% to 5.4%) when testing at 75% relative intensity. The Bland–Altman plots indicated that measurement bias ranged from 6 N to 63 N, consistently favoring trials performed during DESC (Figure 3).

### 3.2. Net Isometric Peak Force at 25% Relative Intensity

Measured and INT Net IPF at 25% relative intensity are presented in Table 3. There was a statistically significant main effect of trial (F_2,68_ = 50.491, *p* < 0.001, η_p_^2^ = 0.598), main effect of sex (F_1,34_ = 31.106, *p* < 0.001, η_p_^2^ = 0.478), and statistically significant interaction effects (trial × sex) (F_2,68_ = 3.359, *p* < 0.05, η_p_^2^ = 0.090) observed for force values at 25% relative intensity. Post hoc testing with Bonferroni adjustment revealed that the isometric force recorded during ASC25 (899 ± 373 N, *p* < 0.001, *g* = 1.38, 0.91 to 1.84) and DESC25 (962 ± 440 N, *p* < 0.001, *g* = 1.42, 0.94 to 1.89) trials were statistically significantly greater compared to INT25 (530 ± 184 N). Net IPF values recorded during DESC25 and ASC25 were not statistically significantly different (*p* = 0.430, *g* = 0.25, −0.09 to 0.59). At 25% relative intensity, Net IPF values were statistically significantly greater for males compared to females (1007 ± 395 N vs. 587 ± 266 N, *p* < 0.001, *g* = 1.34, 0.81 to 1.86).

**Figure 4:**
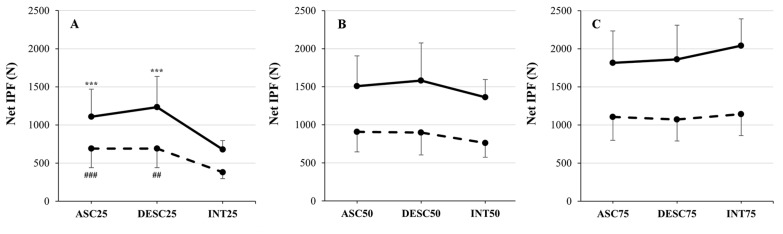
Within-subject comparisons between measured and intended force during ascending (ASC) and descending (DESC) trials at 25% (**A**), 50% (**B**), and 75% (**C**) relative intensity of effort. Solid line = males; dashed line = females. *** indicates statistically significant difference from male INT values at *p* < 0.001. ## and ### indicate statistically significant difference from female INT values at *p* < 0.01 and *p* < 0.001, respectively.

When analyzing trial × sex interaction effects at 25% relative intensity, post hoc testing with Bonferroni adjustment revealed that the Net IPF recorded for males during ASC25 was statistically significantly greater compared to values recorded for females during ASC25 (*p* = 0.004, *g* = 1.37, 0.62 to 2.10), during DESC25 (*p* = 0.009, *g* = 1.28, 0.54 to 2.00), and female INT25 (*p* < 0.001, *g* = 3.19, 2.17 to 4.18). Force values recorded for males during DESC25 were statistically significantly greater compared to values recorded for females during ASC25 (*p* < 0.001, *g* = 1.67, 0.89 to 2.43), during DESC25 (*p* < 0.001, *g* = 1.57, 0.80 to 2.32), and compared to female INT25 (*p* < 0.001, *g* = 3.35, 2.31 to 4.38). Male INT25 was statistically significantly greater than female INT25 (*p* < 0.001, *g* = 2.86, 1.90 to 3.80). Within-subject trial × sex interaction effects observed at 25% relative intensity are presented in Figure 4A.

### 3.3. Net Isometric Peak Force at 50% Relative Intensity

Measured and INT Net IPF at 50% relative intensity are presented in Table 3. There was a statistically significant main effect of trial (F_2,68_ = 8.211, *p* < 0.001, η_p_^2^ = 0.195) and sex observed for force values at 50% relative intensity (F_1,34_ = 43.141, *p* < 0.001, η_p_^2^ = 0.559). Trial × sex interaction effects did not reach statistical significance (F_2,68_ = 0.525, *p* = 0.594, η_p_^2^ = 0.015) (Figure 4B). Post hoc testing with Bonferroni adjustment revealed that Net IPF values recorded during ASC50 (1208 ± 451 N, *p* = 0.009, *g* = 0.54, 0.18 to 0.90) and DESC50 (1239 ± 530 N, *p* = 0.007, *g* = 0.56, 0.19 to 0.91) were statistically significantly greater compared to INT50 (1060 ± 369 N). Forces recorded during DESC50 and ASC50 were not statistically significantly different (*p* = 1.000, *g* = 0.13, −0.20 to 0.47). At 50% relative intensity, isometric force values were statistically significantly greater for males compared to females (*p* < 0.001, *g* = 2.22, 1.37 to 3.07).

### 3.4. Net Isometric Peak Force at 75% Relative Intensity

Measured and INT Net IPF at 75% relative intensity are presented in Table 3. There was a statistically significant main effect of trial (F_2,68_ = 5.750, *p* = 0.010, η_p_^2^ = 0.145) and sex (F_1,34_ = 55.690, *p* < 0.001, η_p_^2^ = 0.621) observed for force values at 75% relative intensity. Trial × sex interaction effects did not reach statistical significance (F_2,68_ = 2.314, *p* = 0.121, η_p_^2^ = 0.064) (Figure 4C). Post hoc testing with Bonferroni adjustment revealed that INT75 (1590 ± 553 N) was statistically significantly greater compared to ASC75 (1459 ± 511 N, *p* = 0.018, *g* = 0.50, 0.14 to 0.85). Differences between INT75 and DESC75 (1465 ± 545 N) approached but did not reach statistical significance (*p* = 0.070, *g* = 0.40, 0.05 to 0.75). Net IPF measured during DESC and ASC trials was not statistically significantly different (*p* = 1.000, *g* = 0.03, −0.30 to 0.37). At 75% relative intensity, Net IPF values were statistically significantly greater for males compared to females (*p* < 0.001, *g* = 2.53, 1.63 to 3.42).

### 3.5. Effects of Testing Order and Relative Error in Force Modulation

The two-way repeated-measures ANOVAs revealed that the main effects of trial and order were not statistically significant at any of the investigated intensities (*p* < 0.05). However, trial × order interaction effects were statistically significant at 25% (F_1,34_ = 8.783, *p* < 0.01, η_p_^2^ = 0.205) and 50% (F_1,34_ = 7.703, *p* < 0.01, η_p_^2^ = 0.185). Post hoc testing with Bonferroni adjustment revealed that relative error was greater during DESC25 compared to ASC25 for subjects who completed DESC25 after maximal-effort testing (26.7 ± 12.7% vs. 18.9 ± 13.0%, *p* < 0.05, *g* = 0.50, 0.14 to 0.85). Furthermore, relative error was greater when DESC25 was performed after rather than before maximal-effort testing (26.7 ± 12.7% vs. 14.3 ± 12.1%, *p* < 0.05, *g* = 1.02, 0.30 to 1.72). At 50% relative intensity, post hoc testing revealed that relative error was also greater for those subjects who completed DESC50 after rather than before maximal-effort testing (14.1 ± 9.0% vs. 2.6 ± 14.7%, *p* < 0.05, *g* = 0.96, 0.25 to 1.66).

### 3.6. Average Relative Error and Sex-Based Differences

**Figure 5:**
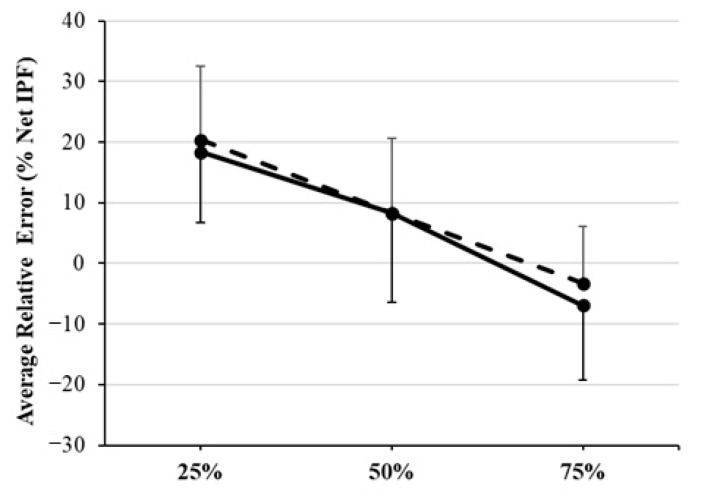
Relative error in force modulation for males and females at 25%, 50%, and 75% net isometric peak force (Net IPF) averaged across ascending and descending trials. Solid line = males; dashed line = females.

The relative error in force modulation observed for the total sample averaged across ASC and DESC trials was 19.4 ± 11.7%, 8.3 ± 13.4%, and −5.1 ± 11.0% at 25%, 50%, and 75% relative intensity of effort, respectively. Independent-samples Student’s *t*-tests revealed that there were no statistically significant differences in the average relative error observed at 25% (20.4 ± 12.1% vs. 18.3 ± 11.6%, *g* = 0.17, −0.49 to 0.82), 50% (8.3 ± 12.3% vs. 8.3 ± 14.7%, *g* = 0.00, −0.65 to 0.65), or 75% (−3.3 ± 9.3% vs. −6.9 ± 12.4%, *g* = 0.33, −0.33 to 0.98) intensity of effort for females and males, respectively (*p* > 0.05) (Figure 5).

### 3.7. Associations Between Subject Relative Strength and Average Relative Error

**Figure 6:**
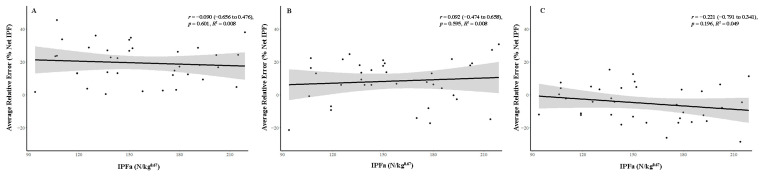
Pearson correlations (*r*) and coefficient of determination (R^2^) reflecting associations and shared variance, respectively, between subject gross isometric peak force allometrically scaled to body mass (IPFa) and average relative error at 25% (**A**), 50% (**B**), and 75% (**C**) relative intensity of effort for all subjects (n = 36). The shaded area indicates the 95% confidence interval after Fisher z transformation.

Statistically non-significant small relationships were observed between subject IPFa and the average relative error observed at every relative intensity investigated (*r* = −0.221 to 0.092, *p* > 0.05) (Figure 6).

## 4. Discussion

This study was conducted to investigate voluntary force modulation accuracy during the IMTP test in strength-trained males and females, to examine potential sex-based disparities in accuracy, and to examine the influence of subject relative strength on the observed error. Submaximal trials were completed in ASC and DESC order before or after maximal-effort testing to enable the identification of effects of testing sequence. External feedback was not provided to subjects during testing, to ensure recorded force values reflected subjects’ perceptions of effort only. The results of this study indicate that, regardless of the relative intensity of effort, error is expected when performing the IMTP test. However, the magnitude of error is greatly reduced when subjects perform the test at moderate-to-high relative intensities (i.e., 50% to 75%). Partially confirming the original hypotheses, subjects overestimated forces at 25% and 50% relative intensity and underestimated force at 75% relative intensity; however, sex-based disparities were not present, and subject relative strength was not strongly associated with the error observed at any intensity.

It has been suggested that the ability to modulate muscular force may align with the central tendency theory as described by Hollingworth [16,39]. According to this premise, subjects are expected to be less accurate when expressing relatively low and high forces, and accuracy is expected to improve near the center of the force spectrum. This phenomenon has been supported [16,40] and refuted [17,18,41,42] by the results of previous research. An alternative hypothesis suggests that force modulation accuracy is expected to decline at greater relative intensities, possibly due to a subconscious protective mechanism that acts to prevent metabolic and mechanical damage in the involved musculature [42]. The results of the current investigation contradict these premises, supporting instead the view that accuracy improves as one approaches one’s capacity for force production.

As the subjects of this study had prior strength training experience, it is possible that repeated practice expressing moderate and high relative intensities during strength training sessions, along with the expected neuromuscular adaptations [5,21,39], contributed to the greater force modulation accuracy observed with increased intensity of effort. This hypothesis is partially supported by the results reported by Rizzato et al. [18] and Majumdar et al. [26], which indicate that trained athletes demonstrate greater accuracy in voluntary force modulation than untrained controls. The authors concluded that the greater accuracy demonstrated by the athletes was likely due to habitual training practices, which may enhance motor unit recruitment, synchrony, and firing rates [21,39], all of which may improve force modulation [5,19,21,43]. However, in contrast to the current study’s findings, Rizzato et al. [18] reported that accuracy decreased as the relative intensity of effort increased when subjects were not provided external feedback, irrespective of subject training status; therefore, additional factors may influence force modulation ability.

The methods of testing used in previous research are important considerations when reconciling the current and previously reported results. The available prior investigations in which force modulation ability was assessed included unilateral and single-joint testing methods [16,17,18,26]. As these actions do not emulate sporting movements, the results of the current investigation may more accurately reflect the force modulation capabilities expected of athletes when performing sport-specific technical movements. It has been suggested that the musculature of the lower body, being predominant in gross movement patterns, may be insensitive to lower relative forces [18,42], which could explain the greater error observed during low-intensity efforts in the current study. Alternatively, the error observed during low-intensity efforts may be attributed to IMTP testing methods, which require subjects to express a minimal amount of force (i.e., 100 ± 100 N) to remove the slack from the system immediately prior to the onset of each trial [2]. However, the tendency to overestimate force was not observed at 75% relative intensity, suggesting that subjects may be better able to perceive greater relative intensities, regardless of the initial force expression required at the onset of testing.

Evidence from prior investigations indicates that force modulation accuracy may be influenced by the type and duration of contraction performed. Miyamoto et al. [17] and Rizzato et al. [18] found that ballistic contractions resulted in greater overall accuracy compared to isometric contractions. The researchers attributed the discrepancies to greater motor unit recruitment and firing rates during ballistic contractions, as well as additional afferent feedback provided during isometric contractions of relatively greater duration, which may have influenced perceptions of effort [17,18]. Conflicting results have been reported when isometric contractions were investigated, with Keller et al. [16] reporting superior accuracy at moderate intensities (30% to 70% MVC) and Miyamoto et al. [17] and Rizzato et al. [18] reporting greater accuracy when three-second contractions were performed at low intensities (20% and 25% MVC, respectively). Although the duration of isometric contractions was not specified in the former study [16], it is conceivable that differential sensory feedback corresponding to differing contraction durations may partially explain the conflicting results. During the IMTP test, subjects in the current study performed brief isometric contractions (one to three seconds), minimizing the time during which sensory information was received. As both the current and prior investigations examined isometric contractions of similar duration [17,18], alternative factors may explain the discrepancies in the results reported.

In the present study, males and females demonstrated identical patterns of error, overestimating low and moderate relative forces and underestimating high relative forces. These results are in partial agreement with those reported by Keller et al. [16], where male and female subjects overestimated forces at low relative intensities (10% and 30%) and underestimated forces at high relative intensities (70% and 90% MVC), although males tended to demonstrate poorer accuracy. It appears that the difference in relative error observed for males and females at low, moderate, and high relative intensities has not been reported in previous research. In the current study, differences in error between males and females at low (+2.1%), moderate (+0.0%), and high relative intensities (−3.6%) were not statistically significant and have questionable practical significance, evidenced by trivial-to-small between-group effect sizes.

It has been hypothesized that force modulation accuracy may be inversely related to total muscle mass and force production capacity, and that this relationship may contribute to sex-based disparities in force modulation capabilities [16,42]. Although males in the current study demonstrated greater Net IPF during maximal-effort testing and greater Net IPF at every relative intensity investigated, similar error was observed for both sexes, suggesting that absolute maximal muscular strength may not be negatively associated with force modulation accuracy. It is possible that the methods used to investigate force modulation in this study partially obfuscated sex-based physiological differences, as the force of subject body mass was removed from each isometric force measurement prior to analysis. After adjusting for subject body mass, the results presented here suggest that biological sex does not appear to influence force modulation accuracy during the IMTP test.

Although associations between maximal force capacity and technical sport-specific skill performance have been investigated previously [12,13,14,15], to the author’s knowledge, this is the first study investigating associations between subject relative strength and force modulation accuracy during a general physical test. The results of the current study indicate that relative strength is not strongly associated with force modulation accuracy, suggesting that alternative factors, such as acute perception [16,18] and greater familiarity through practice [18], may explain individual differences.

In prior investigations, researchers have examined force modulation accuracy after subjects were provided with low and high reference forces, generally in the form of complete muscular relaxation followed by an MVC performed prior to submaximal testing [16,17,18,25,42]. This strategy, often referred to as anchoring [16,25,42], has been purported to enhance force modulation accuracy by guiding subjects’ perceptions of effort during submaximal testing [16,25]. Evidence supporting anchoring effects is lacking and inconsistent [16,18,41]; as such, the efficacy of anchoring strategies in enhancing force modulation is currently inconclusive. In the current investigation, the lack of statistically significant improvement in relative error observed following maximal-effort testing indicates a lack of positive anchoring effects. However, at 25% and 50% relative intensity of effort, differences in error were detected between trials completed before or after maximal-effort testing. Interestingly, rather than supporting the purported improvements in accuracy, the results of the current study suggest that performing an MVC prior to submaximal testing may increase relative error. An alternative explanation for these results may be that subjects accumulated fatigue during the single testing session after performing multiple submaximal and maximal-effort IMTP trials. However, the lack of statistically significant differences observed at 75% intensity between trials performed before or after maximal-effort testing indicates that fatigue effects may not have contributed to these results. Therefore, it is plausible that the provision of a reference MVC prior to submaximal testing may skew the subject’s perception of lower relative forces.

This study is not without limitations. Subjects varied in their prior experience with the IMTP test, and time constraints permitted only minimal familiarization prior to data collection. Nevertheless, all subjects had prior experience performing weightlifting derivative exercises and, although differences were observed in the average relative error for those subjects with (n = 12) and without (n = 24) prior IMTP test experience at 25% (22.7 ± 12.6% vs. 17.7 ± 11.2%, *g* = 0.42, −0.28 to 1.12), 50% (11.7 ± 12.1% vs. 6.6 ± 13.9%, *g* = 0.38, −0.33 to 1.07), and 75% (−4.5 ± 12.4% vs. −5.4 ± 10.4%, *g* = 0.08, −0.61 to 0.77) relative intensity of effort, these differences may not be practically significant. Although an inferential analysis was not possible, due to small and unbalanced samples, the relatively small differences in the average relative error observed, as well as the identical pattern in error observed across the relative intensities investigated, suggest that only minimal prior experience performing the IMTP test may be required to achieve results similar to those reported here. Additionally, the subjects of this study were strength-trained, as are most athletes; however, subjects were not expected to have been trained in performing technical, sport-specific skills requiring movement precision and the careful application of submaximal muscular forces. Although many of the subjects in the current investigation were current athletes competing in a variety of sports in the National Collegiate Athletic Association (NCAA) at the Division I level (wrestling: 1; American football: 2; volleyball: 1; track and field: 4; tennis: 1; baseball: 3; rowing: 3; soccer: 1), most were non-competitive strength-trained individuals (n = 20). As prior training involving the expression of force at a given relative intensity appears to be a crucial factor influencing force modulation accuracy, the results presented here may not reflect the capabilities of athletic populations who practice performing sport-specific technical skills that require modulation of lower relative forces (e.g., ≤25% MVC).

## 5. Conclusions

The IMTP test is a viable tool for assessing IPF and force modulation accuracy at moderate and high relative intensities; however, subjects appear to greatly overestimate force at low relative intensities. Therefore, the IMTP test may not be appropriate for assessing force modulation capabilities at lower intensities. Furthermore, subject relative strength is not strongly associated with force modulation accuracy; rather, habitual training practices involving the expression of specific relative intensities to be assessed may be a more dominant factor influencing force modulation accuracy. Practitioners should prepare and monitor athletes when modulating muscular force in a manner that emulates force requirements for tasks performed during competition. Practitioners may use the IMTP test to examine force modulation abilities in both male and female subjects, as accuracy does not appear to be influenced by biological sex. Finally, the results presented here indicate that only minimal prior familiarization with the IMTP test may be required for subjects to demonstrate force modulation abilities similar to those with greater prior familiarization, although this observation requires further investigation.

## Figures and Tables

**Table 1:** Subject descriptive characteristics.

	Total Sample (n = 36)	Males (n = 18)	Females (n = 18)
Age (yrs)	23.7 ± 5.2	24.8 ± 5.8	22.7 ± 4.4
Training Age (yrs)	7.2 ± 4.7	8.3 ± 5.0	6.2 ± 4.4
BM (kg)	76.3 ± 12.0	84.3 ± 10.0	68.4 ± 7.9
Ht. (m)	1.70 ± 0.09	1.75 ± 0.07	1.65 ± 0.07
IPF (N)	2869 ± 817	3546 ± 505	2192 ± 385
IPFa (N/kg^0.67^)	156.0 ± 35.1	182.2 ± 23.5	129.8 ± 23.0

Note: BM = body mass; Ht.= height; IPF = gross isometric peak force; IPFa = gross isometric peak force allometrically scaled.

**Table 2:** Measurement reliability of net isometric peak force data.

ICC_3,1_			
	Total Sample	Males	Females
25%	0.850 (0.726 to 0.921)	0.765 (0.474 to 0.905)	0.682 (0.328 to 0.868)
50%	0.908 (0.828 to 0.952)	0.802 (0.545 to 0.921)	0.820 (0.582 to 0.929)
75%	0.952 (0.909 to 0.975)	0.897 (0.747 to 0.960)	0.862 (0.668 to 0.946)
100%	0.994 (0.989 to 0.997)	0.981 (0.951 to 0.993)	0.983 (0.954 to 0.993)
CV			
	Total Sample	Males	Females
25%	8.4 (6.5 to 10.3)	7.1 (4.8 to 9.4)	9.7 (6.5 to 12.9)
50%	6.6 (5.1 to 8.1)	6.4 (4.3 to 8.5)	6.9 (4.6 to 9.2)
75%	5.0 (3.8 to 6.2)	4.7 (3.2 to 6.2)	5.4 (3.6 to 7.2)
100%	1.7 (1.3 to 2.1)	1.5 (1.0 to 2.0)	1.9 (1.3 to 2.5)

Note: ICC_3,1_ = intraclass correlation coefficient; CV = coefficient of variation; % = % of maximal effort.

**Table 3:** Net isometric peak force data and corresponding effect sizes.

	Peak Force (N)			Effect Sizes (Hedges’ *g*)		
	ASC25	DESC25	INT25	ASC25 vs. DESC25	ASC25 vs. INT25	DESC25 vs. INT25
M	1108 ± 361	1232 ± 405	680 ± 117	−0.35 (−0.69 to −0.01)	1.14 (0.70 to 1.56)	1.29 (0.83 to 1.73)
F	690 ± 251	692 ± 284	380 ± 94	−0.01 (−0.34 to 0.33)	0.82 (0.43 to 1.20)	0.73 (0.35 to 1.10)
	ASC50	DESC50	INT50	ASC50 vs. DESC50	ASC50 vs. INT50	DESC50 vs. INT50
M	1507 ± 399	1580 ± 495	1359 ± 235	−0.22 (−0.56 to 0.12)	0.38 (0.04 to 0.73)	0.49 (0.13 to 0.83)
F	908 ± 265	898 ± 294	761 ± 188	0.03 (−0.30 to 0.36)	0.38 (0.04 to 0.72)	0.30 (−0.04 to 0.64)
	ASC75	DESC75	INT75	ASC75 vs. DESC75	ASC75 vs. INT75	DESC75 vs. INT75
M	1815 ± 420	1860 ± 451	2039 ± 352	−0.18 (−0.51 to 0.16)	−0.60 (−0.96 to −0.24)	−0.41 (−0.75 to −0.06)
F	1104 ± 304	1071 ± 282	1141 ± 282	0.13 (−0.21 to 0.46)	−0.10 (−0.43 to 0.23)	−0.16 (−0.49 to 0.18)

Note: Net isometric peak force data presented as mean ± standard deviation (N). Effect sizes presented as Hedges’ *g*. M = male; F = female; ASC25 = ascending 25%; DESC25 = descending 25%; INT25 = intended force at 25%; ASC50 = ascending 50%; DESC50 = descending 50%; INT50 = intended force at 50%; ASC75 = ascending 75%; DESC75 = descending 75%; INT75 = intended force at 75%.

## Data Availability

Data are available upon reasonable request.
